# Sea level rise outpaced by vertical dune toe translation on prograding coasts

**DOI:** 10.1038/s41598-021-92150-x

**Published:** 2021-06-17

**Authors:** Christa O. van IJzendoorn, Sierd de Vries, Caroline Hallin, Patrick A. Hesp

**Affiliations:** 1 Faculty of Civil Engineering and Geosciences, Delft University of Technology, Delft, The Netherlands; 2 Division of Water Resources Engineering, Faculty of Engineering, Lund University, Lund, Sweden; 3 Beach and Dune Systems (BEADS) Laboratory, College of Science and Engineering, Flinders University, Bedford Park, SA 5041 Australia

**Keywords:** Geomorphology, Ocean sciences, Climate change, Civil engineering

## Abstract

Sea level is rising due to climate change and is expected to influence the development and dynamics of coastal dunes. However, the anticipated changes to coastal dunes have not yet been demonstrated using field data. Here, we provide evidence of dune translation that is characterized by a linear increase of the dune toe elevation on the order of 13–15 mm/year during recent decades along the Dutch coast. This rate of increase is a remarkable 7–8 times greater than the measured sea level rise. The observed vertical dune toe translation coincides with seaward movement of the dune toe (i.e., progradation), which shows similarities to prograding coasts in the Holocene both along the Dutch coast and elsewhere. Thus, we suspect that other locations besides the Dutch coast might also show such large ratios between sea level rise and dune toe elevation increase. This phenomenon might significantly influence the expected impact of sea level rise and climate change adaptation measures.

## Introduction

Coastal dunes are of vital importance for coastal protection and flood safety, have high geomorphological, ecological and intrinsic values, and are an important recreational resource along large parts of the world’s coasts. Climate change, and its associated sea level rise (SLR), is an important driver for the development of coastal dunes. Numerous studies have investigated the coastline response to sea level rise^1–8^, but it remains uncertain how SLR is impacting coastal dunes. Most predictions of coastline change due to SLR^4–6^ are based on variations of the Bruun rule^7^. The Bruun rule assumes that with rising sea level the beach is eroded and that the eroded sediment is moved to the offshore part of the coastal profile^7^. Work that expands on the Bruun rule predicts that, with SLR, the beach and dune will maintain their shape while moving landward and upward^8,9^. This implies that the dune toe will show the same behaviour. Here, the dune toe is defined as the boundary between the backshore limit (commonly around the high spring tide limit) and the seaward edge of the dunes.

The dune toe is a key parameter to describe the dune profile and is part of the spatially and temporally varying beach-dune system typically moving seawards during accretionary periods, and landwards during storms and erosion events^10–13^. With SLR, the vertical extent of marine processes is altered, which influences the sediment budget of sandy beaches. Thus, it can be expected that SLR influences both the horizontal and the vertical dune toe position^14^. In this research, we present a unique study that compares the response of coastal dunes to sea level rise by tracking the horizontal and vertical dune toe position in an extensive dataset with measured coastal profiles.

In the Netherlands, coastal dune-beach-nearshore profiles have been measured yearly since 1965 (i.e., the Jarkus dataset). This is the largest dataset of measured coastal profiles in the world and it has been used to analyse decadal morphological development in several previous studies^15–17^. However, the vertical translation of the dune toe has not previously been investigated. Traditionally, the vertical location of the dune toe in the Netherlands has been defined as a temporal constant, namely as the point in a beach-dune profile at 3 m elevation above NAP (Normaal Amsterdams Peil—the Dutch fixed reference datum)^18^. This traditional method to identify the dune toe location is easy to apply and suitable for short-term applications. However, it cannot be used to track the changes in the dune toe elevation that occur due to longer-term natural variations of the dune profile. More advanced methods include defining the dune toe position as the location of maximum slope change that occurs landward of the shoreline^19–23^ or calculating the dune toe position based on the sediment volume between + 1 and + 5 m NAP^24^. Alternatively, in natural dune systems, the dune toe can be based on the seaward extent of dune vegetation^14,25^ since the vegetation cannot grow seawards of the high spring tide line and semi-regular tidal inundation. The application of the different dune toe identification methods depends on the context in which they are used, the available data and the preference of the user^26,27^.

**Figure 1:**
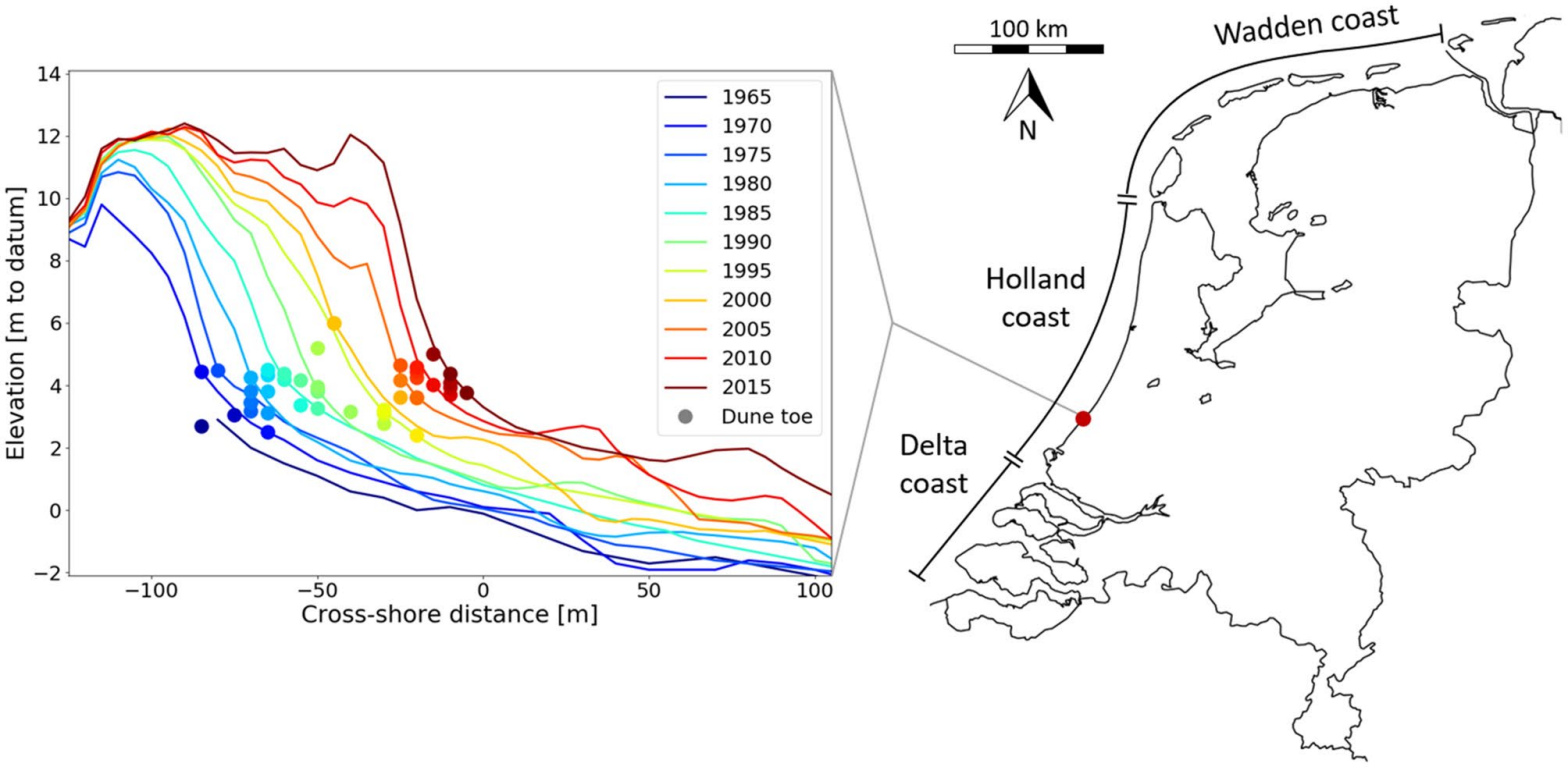
Example of dune toe identification for a transect (ID. 9010235) along the Holland Coast. Colored lines show the profiles measured between 1965 and 2005, for clarity displayed with a 5-year interval, revealing the morphological development through time. Colored dots represent the extracted dune toe position for each year in the period from 1965 (dark blue) to 2017 (dark red). For some years an indication of the dune toe is missing because the algorithm was not able to extract a dune toe from the corresponding profile. The map in this figure was created using basemap 1.2.0 (https://matplotlib.org/basemap/index.html).

Recently, new semi-automated methods have become available that can systematically extract the dune toe position from coastal profiles^28–33^. These new methods strive towards providing a generic derivation of a dynamic dune toe. A generic definition of the dune toe, consistent through space and time, makes it possible to track temporal and spatial variations in the dune toe position on an unprecedented scale using large datasets. It should be noted that the available methods have varying degrees of subjectivity depending on user input^26^. The results presented in this research were obtained by analysing dune toes extracted with the second derivative method^32^ from the Dutch Jarkus dataset. Additionally, the machine learning method of *pybeach*^31^ was used to verify that a different extraction method gives similar results. The second derivative method was developed and tested by Diamantidou et al. (2020) using the Jarkus dataset. A comparison with in situ visual observations showed that the method selects the dune toe consistently^32^. An example of measured profiles and derived dune toes is presented in Fig. 1.

## Increase in elevation and seaward movement of the dune toe

**Figure 2:**
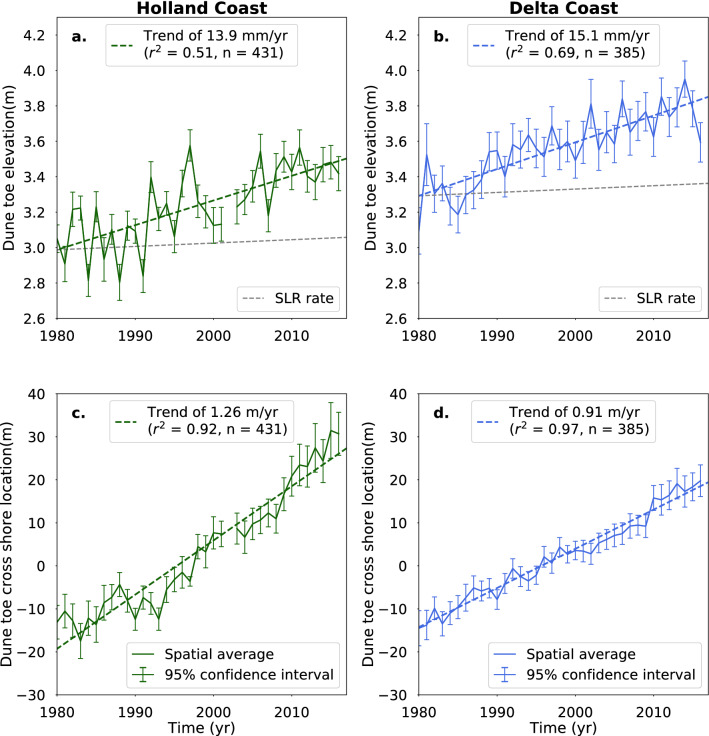
Trends in the dune toe position along the Holland coast (**a**, **c**) and Delta coast (**b**, **d**). The trend in dune toe elevation (**a**, **b**), and the trend in cross-shore dune toe location (**c**, **d**) are shown. In each subplot, the spatial average of all transects along the coast is represented by the solid line. The vertical bars along this line show the 95% confidence interval for each year. The overall trend in the spatial average is represented by the dashed line. In each subplot, the rate (in m/year), r-squared value and number of transect locations (n) of this trend are given in the upper left corner. The grey dashed lines in subplots (**a**, **b**) show the development of the dune toe elevation if it had increased at the same rate as sea level rise (SLR).

Between 1980 and 2017, the derived dune toe elevation along the Holland coast shows a linear increase in time of 13.9 mm/year (r^2^ = 0.51) and along the Delta coast, an increase of 15.1 mm/year (r^2^ = 0.69) (Fig. 2). The derived dune toe position along the Holland and Delta coast also shows seaward movement of 1.26 m/year (r^2^ = 0.92) and 0.91 m/year (r^2^ = 0.97), respectively. At the Wadden coast, no significant trend in the data was found. The regional morphological variability associated with the tidal inlets in this area likely dominates the decadal changes in the dune toe position. The trends shown in Fig. 2 are based on nearly 27.000 dune toe positions spread over 816 different transects. The derived dune toe elevations for both the Holland and Delta coast vary within a range of 2–4 m + NAP, which includes the commonly assumed value of ~ 3 m + NAP. Similar results are found using dune toes derived with the machine learning method of *pybeach* (Supplementary Figure 1).

## Physical processes explain regional variations in dune toe position

**Figure 3:**
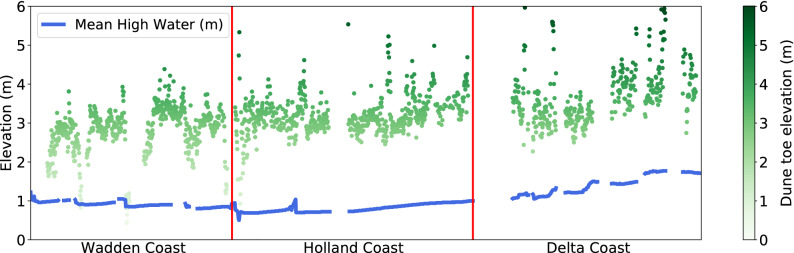
Alongshore variation of the temporally averaged dune toe elevation (m) for the period between 1980 and 2017. Each dot (in green) represents the average value for a transect along the Dutch coast. The blue line shows the alongshore variation in the mean high water as included in the Jarkus dataset.

Alongshore variations in the dune toe elevation are probably related to alongshore variations in waves and tides. Other mechanisms which might be related are, e.g., geological framework, grain size variations and vegetation dynamics; however, these are relatively uniform along the Dutch coast. The dune toe elevations along the Delta coast are overall higher than along the Holland coast (Fig. 3), which is expected based on the higher mean high water levels along the Delta coast which lead to higher extreme water levels. Along the Delta coast, the dune toe elevation increases by approximately 1.0 m in a southward direction, while the mean high water also shows an increase in elevation in that direction. A similar relation is visible for the southern part of the Holland coast. However, along the entire Dutch coast, dune toe elevations are between 0.5 and 3.0 m higher than the mean high water. This difference may be explained by the variations in wave exposure that are superimposed on the tides. For instance, dune toe elevations peak in the centre of the islands along the Delta and Wadden coast, where waves arrive perpendicular to the coast. In contrast, dune toe elevations are lower near the edges of the islands, where wave exposure is expected and likely to be less.

These findings are in line with the common assumption that the vertical location of the dune toe is mostly dependent upon marine processes. For instance, the dune toe position has been related to the maximum total water level (TWL) that occurs every ten years^22^. The TWL is made up of the measured tide level, setup and swash^34^. The factors influencing the TWL, and thus possibly the dune toe elevation, can be summarized as (1) waves, (2) astronomical and meteorological tides and (3) mean sea level. The spatial and temporal resolution of water level and wave height data was not sufficient to correlate the TWL to the dune toe elevation in this study. Still, in Fig. 3, we probably see the effect of waves and tides causing alongshore variation in dune toe elevation. We cannot see the effect of mean sea level because that does not have significant variations on this spatial scale. Temporal variations in sea level might, however, be linked to the increase of the dune toe elevation through time.

## Dune translation outpaces sea level rise

**Figure 4:**
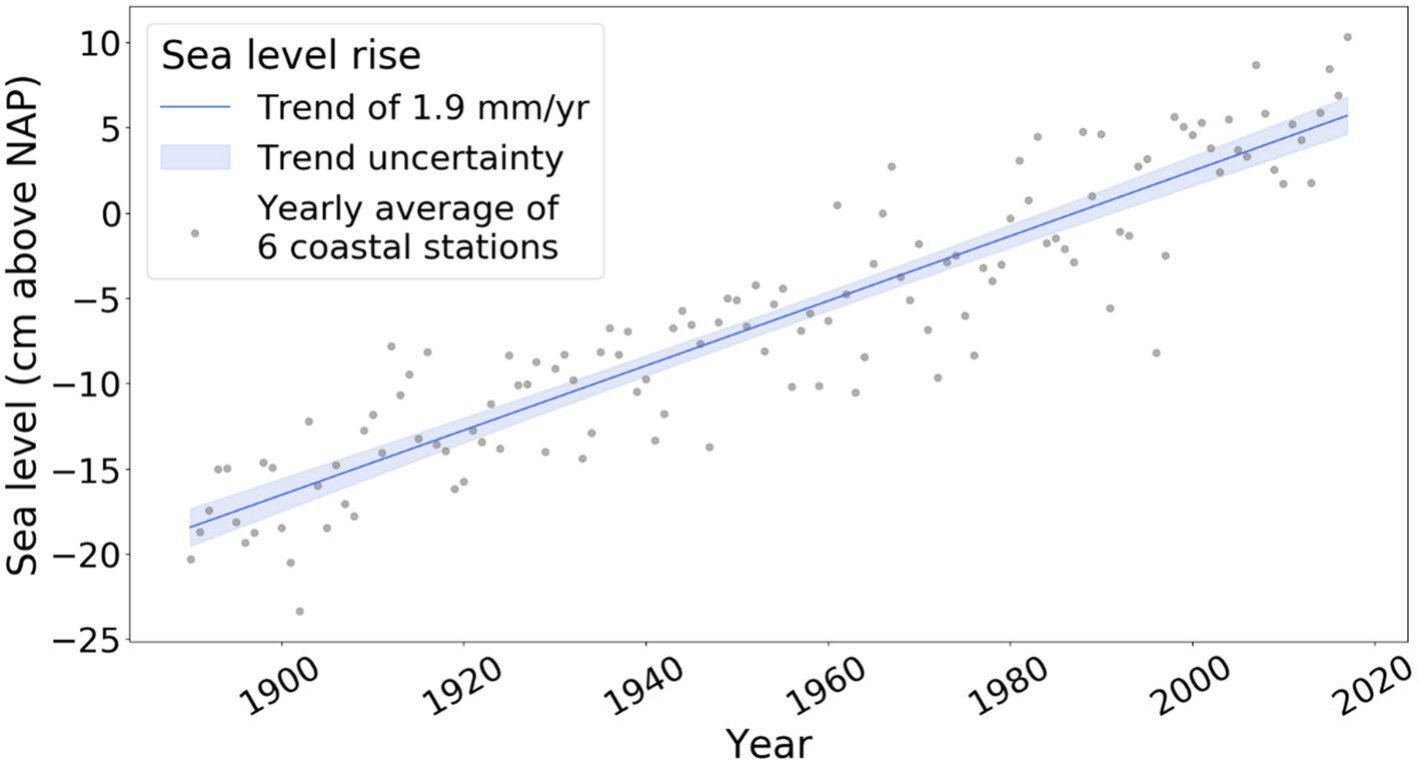
Sea level rise along the Dutch coast measured at 6 different coastal stations from 1880 to 2014^35^. Each grey dot represents the yearly averaged value of the 6 coastal station. The solid blue line represents the trend in these yearly averaged values and the uncertainty of this trend is represented by the blue shading. This uncertainty was calculated as the trend ± 2 standard deviations.

The SLR trend along the Dutch coast is linear and consistent at a rate of 1.9 mm per year (Fig. 4)^35^. Conceptual models based on the Bruun rule assume a 1:1 ratio between sea level rise and dune toe elevation increase^8,36^. Our results show that this assumption is not valid for the Holland and Delta coast. SLR is significantly slower than the increase in dune toe elevation (Fig. 2), namely 1.9 mm/year SLR compared to 13.9–15.1 mm/year dune toe elevation increase. We may define this difference between the rate in dune toe elevation increase and SLR as the Dune Translation Index (DTI). The DTI is derived by dividing the rate of dune toe change by the mean SLR, which in the case of the Holland coast results in a DTI of 7.33, and for the Delta coast, a DTI of 7.95. Thus, for the Holland and Delta coast together, the mean DTI is estimated to be 7.6. At this moment, we cannot fully explain the physics behind the DTI. However, as additional data from other coasts become available, the DTI provides a means by which to compare the relationship between SLR and vertical dune toe translation for different surfzone-beach types and various retrogradation to progradation (i.e., landward to seaward movement) rates.

## Similarities to Holocene transgression

The contemporary behaviour of the dune toe position along the Dutch coast is similar to its documented seaward movement during SLR in the Holocene^37^ and to Holocene coastal barrier development at other locations. At a millennium scale, several studies of coastal barriers show that as the Holocene sea level was rising in the 8000 to 5000 years BP period, where there was sufficient sediment supply, barriers (dunes, beaches and shoreface) prograded seawards in concert with the backshore/dune contact rising vertically^38–40^. This same increase in elevation and progradation of the coastal profile also occurred along the Dutch coast during the Holocene^37^. The progradation of the profile was linked to the supply of sediment from offshore (i.e., shoreface feeding)^41^ while rising sea level caused the dune toe elevation to increase. Currently, the dune toe is increasing in elevation and prograding along the Holland and Delta coast (Fig. 2). Based on this study, it is uncertain whether the entire dune is prograding in relation to SLR simultaneously with the dune toe. This depends on the behaviour of the dune crest and the associated profile shape. Milder dune slopes will develop over time if dune crests are less mobile than the dune toe. Since this is the first study of its kind, it remains unclear if the contemporary changes in the dune toe position along the Dutch coast also occur at other locations. If that is the case, we might expect other locations where the dune toe elevation increase outpaces SLR.

## Vertical dune toe translation 7–8 times faster than SLR

The DTI of 7.6 shows that vertical dune toe translation (14–15 mm/year) outpaces SLR (1.9 mm/year) along the Dutch coast with a remarkable rate compared to the 1:1 ratio predicted by models based on the Bruun rule^7^. Additionally, the progradation of the dune toe deviates from the seaward movement assumed by the Bruun rule. Initially, when trying to explain the high DTI, it should be considered that this is the first time this extensive dataset has been utilized to determine the relationship between contemporary dune toe elevation and SLR. Thus, the DTI of 7.6 might be the natural rate of change for the surfzone-beach systems typical for the Dutch coast over the past decades. However, the dune toe development cannot continue like this indefinitely because the vertical dune toe position remains related to marine processes. Thus, the dune toe cannot increase to an elevation that is outside of the reach of the total water level.

Future work could include several different aspects that might be able to explain the high DTI. Firstly, the development of the coastal profile could lag behind the changes in SLR conditions, so the equilibrium profile that corresponds to a 1:1 ratio is not reached. Secondly, the influence of other factors besides SLR, like marine and sedimentary processes, should be considered. For instance, temporal variations in the wave climate and tidal range could influence and accelerate an increase in dune toe elevation. Nourishments and vegetation dynamics could change the response of the dune toe on a yearly to decadal-scale by supplying to and trapping sediment at the dune toe. The effect of nourishments might be especially relevant for the Dutch coast considering the large number of nourishments that are applied along the coast^42,43^. Specifically, previous research has related nourishments to a reduction of transects with landward dune toe movement^44^. Additionally, alongshore transport could also influence the dune toe development, but these influences are minor on the national scale.

For now, the measured deviation between sea level and dune toe elevation change cannot be explained in detail because not all physical processes that influence coastal profile change are fully understood. However, the significant deviation from the 1:1 ratio indicated by the high DTI has large implications for predicting the impact of climate change. Since the dune toe position significantly impacts the dune volume, it will also influence the safety standard of dunes and the basis for spatial planning in the coastal zone. Thus, this finding may change the evaluation, implementation, and planning of future climate change adaptation measures along the coast.

## Consequences of the dune toe elevation outpacing SLR

Based on the results, we conclude that along the Holland and Delta coast the increase in dune toe elevation (14–15 mm/year) outpaces SLR (1.9 mm/year) by a factor of 7.6, and the dune toe is moving seaward at 1 m/year. This shows that the decadal-scale behaviour of the coastal profile can strongly deviate from the expected landward horizontal movement and the 1:1 relation with SLR for vertical movement as assumed by some authors^6,7^. We suggest that, based on similar transgressive behaviour in the Holocene, such large ratios might also occur at other locations besides the Dutch coast. If this applies to other coasts, the outpacing of SLR by vertical dune toe translation might significantly alter the predictions of the impact of climate change on coastal dunes. Thus, this finding may change the evaluation, implementation and planning of future climate change adaptation measures along the coast.

## Methods

The dataset with yearly profile measurements (i.e., the Jarkus dataset) used in this research has been collected from 1965 onwards. The transect locations span the entire Dutch coast and are spaced approximately 250–500 m apart. To study the trend in decadal dune toe position the following steps were executed: (1) choosing the dune toe position definition and extraction method, (2) extracting the dune toe positions from the coastal profiles and (3) spatial and temporal filtering of the resulting dune toe positions.

In this research, the dune toe was defined as the boundary between the landward limit of the beach (the top of the backshore) and the dunes. We assumed that the specific location of the dune toe is subordinate to its function as a proxy for coastal profile change. The identification of the dune toe for one individual transect does not have value because there is no ‘true’ dune toe location that is directly related to the physical processes that shape the coastal profile. However, when applying the extraction method to a long-term and/or spatially extensive dataset, we are no longer studying the specific dune toe position, but the changes in the position. Thus, the analysis of the long-term change in dune toe position was based on the assumption that the derivation methods can track the development of the dune toe position consistently.

In choosing an automated extraction method for this research, two criteria were used. First, the method should be open-source to allow for public availability of the research results. Second, the method should be compatible with the Jarkus dataset that contains profile measurements of coastal morphology. Therefore, methods based on digital elevation models (DEMs)^28–30^ and vegetation^14,25^ were excluded. Applicable methods include those based on profile slope^19–23,32^, profile volume^24^ and profile shape^33^. Among the slope-based methods, which are most commonly used, the second derivative method was selected. In a previous study, this method was applied to the Jarkus dataset and showed robust results compared to in situ visual observations^32^. Additionally, the recent, innovative and well-documented *pybeach* method based on machine learning was added to diversify the analysis in an effort to increase the confidence in the observed dune toe development trends.

The second derivative method consists of two main steps. First, the coastal profile is reduced in length with the landward and seaward boundaries depending on the primary dune height and mean high water, respectively. Second, the dune toe is placed at the most seaward location where the first and second derivative of the remaining profile are each larger than their predefined threshold. Thus, some subjectivity is introduced in this method when the boundaries and thresholds are defined^26^. A detailed description of the second derivative method and its application to the Jarkus dataset is provided by Diamantidou et al.^45^ together with a dataset consisting of the extracted dune toes. The *pybeach* method extracts the dune toe using machine learning, where the dune toe identifier is based on dune toe identification executed by experts^31^. This method was applied to the same reduced profile as the second derivative method. Given the resolution of the Jarkus dataset (i.e., 5 m) and the extraction technique of both methods, the derived dune toe positions are not sensitive to smaller-scale variations in the coastal profile. The extracted dune toe locations were filtered to remove years and locations with unreliable data or a lack of data availability. The majority of removed transects were deviating coastal profiles that include, for instance, dikes and dams. These transects (± 23% of all transects) were removed from the dataset leaving 77% of the available data points (about 60.000 dune toe positions) for further analysis.

To obtain an indication of the overall profile evolution of the Dutch coast, the average elevation and cross-shore location of the dune toe of all transects per year was calculated. For the period before 1980, the lack of data for a large number of transects resulted in the average values being skewed. The period between 1980 and 2017 has the most reliable data availability and was thus used for the trend analysis shown in Fig. 2, similar to earlier approaches that used the Jarkus dataset^15^.

The results shown in Figs. 2 and 3 are the output of the second derivative method, whilst the results of the *pybeach* method are shown in Supplementary Figure 1. Overall, the results of the *pybeach* method were similar with a DTI of 6.6 for the Delta and Holland coast combined, compared to a DTI of 7.6 for the second derivative method. The comparable results between the second derivative method and the machine learning method of *pybeach* indicate that the results converge for two different methods when applied to a large dataset. It is expected that other methods based on the profile slope^19–23,32^ would generate similar results. However, methods that use distinctly different calculation techniques, like those based on volume^24^, profile shape^33^ and, especially, based on vegetation^14,25^ might show varying results and would be interesting to explore in future studies.

## Supplementary Information

Supplementary Information.

## Data Availability

The software used to extract and analyse coastal profile parameters from the Jarkus dataset (i.e., the Jarkus Analysis Toolbox), and the dataset containing the extracted parameters that were generated during this research are available in the 4TU repository, https://doi.org/10.4121/c.5335433.
