# Supplementary material for: Sea level rise outpaced by vertical dune toe translation on prograding coasts
Source: Sci Rep. 2021 Jun 17;11:12792. doi: 10.1038/s41598-021-92150-x (PMC8211745; doi:10.1038/s41598-021-92150-x)
Supplement: Supplementary file 1 — Supplementary Information. [file 41598_2021_92150_MOESM1_ESM.docx]

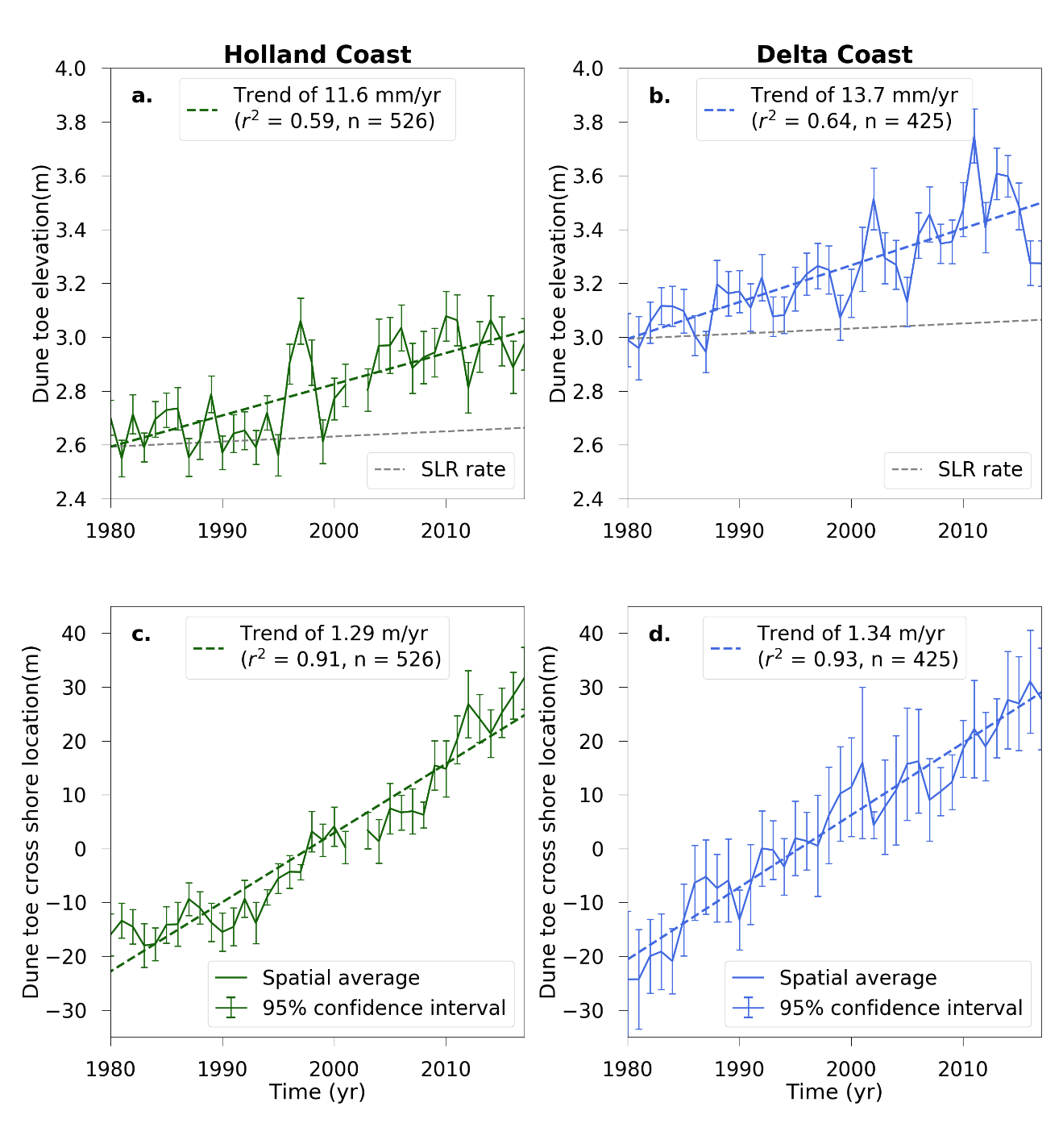


**Sea level rise outpaced by vertical dune toe translation on prograding coasts**
Christa O. van IJzendoorn, Sierd de Vries, Caroline Hallin, Patrick A. Hesp

***Supplementary Figure 1: Trends in the dune toe position along the Holland coast (a and c) and Delta coast (b and d) extracted using the machine learning method of pybeach.*** *The trend in dune toe elevation (a and b), and the trend in the cross-shore dune toe location (c and d) are shown. In each subplot, the spatial average of all transects along the coast is represented by the solid line. The vertical bars along this line show the 95% confidence interval for each year. The overall trend in the spatial average is represented by the dashed line. In each subplot, the rate (in m/yr), r-squared value and number of transect locations (n) of this trend are given in the upper left corner. The grey dashed line in subplots a and b show the development of the dune toe elevation if it had increased at the same rate as sea level rise (SLR).*
